# A Strategic Design of an Opto-Chemical Security Device with Resettable and Reconfigurable Password Based Upon Dual Channel Two-in-One Chemosensor Molecule

**DOI:** 10.1038/srep42811

**Published:** 2017-02-20

**Authors:** Tapas Majumdar, Basudeb Haldar, Arabinda Mallick

**Affiliations:** 1 Department of Chemistry, University of Kalyani, Nadia, West Bengal-741222, India; 2 Department of Chemistry, Vivekananda Mahavidyalaya, Burdwan, Westbengal-713103, India; 3 Department of Chemistry, Kashipur Michael Madhusudan Mahavidyalaya, Purulia, Westbengal-723132, India

## Abstract

A simple strategy is proposed to design and develop an intelligent device based on dual channel ion responsive spectral properties of a commercially available molecule, harmine (HM). The system can process different sets of opto-chemical inputs generating different patterns as fluorescence outputs at specific wavelengths which can provide an additional level of protection exploiting both password and pattern recognitions. The proposed system could have the potential to come up with highly secured combinatorial locks at the molecular level that could pose valuable real time and on-site applications for user authentication.

Development of data processing and information protection platforms using individual molecule mimicking the operation of electronic logic gates have attracted considerable interest in the recent times^1^,^2^,^3^,^4^,^5^,^6^,^7^,^8^,^9^,^10^,^11^,^12^. Lately, scientists have devoted immense effort to build molecular scale logic devices which are capable of processing data and provide protection via authorizing password entries^1^,^2^,^3^,^4^,^5^,^6^,^7^,^8^,^9^,^10^,^11^. In the molecular recognition arena, a number of research groups have used and proposed wide range of molecules, such as diarylethne^13^, benzothiphne^14^, azobenzene^15^ based molecule, BODIPY scafolds^11^, dithienylethene & fulgimides^8^, bipyridyl dio^10^ and so forth, executing different functions mimicking the conventional logic gates such as half-adder^10^, encoders, decoders^16^,^17^, half subtractors^17^, keypad locks^2^,^4^,^18^,^19^,^20^ etc. In recent years, such decision making molecular systems have been explored through tricky applications taking the advantages of binary and multivalued information processing with more complicated functions^8^. Concept of molecular logic gate based fluorescence signalling was first inrouduced by De Silva^2^. Since then a good number of molecular scale combinatorial locks have been developed and parallely, biomolecular keypad locks using enzymes^21^, DNA^22^, aptamers^23^, antibodies^24^ were developed demonstrating with several input channels and a single output channel.

However, in the literature among the significant numbers of available molecular keypad locks, systems with multiple optochemical inputs and multiple optical outputs providing sensitive pattern recognition remained extremely rare. As a result, most of the reported systems are limited to recognize only a single password and practically operable only in one particular solvent system. One approach to circumvent this problem is functional integration of different medium in the targeted synthetic process. Walking ahead along this line, we demonstrate herein, the feasibility of creating a novel class of opto-chemical molecular keypad lock with multiple input sensitivity and dual channel fluorescence outputs with password and pattern recognition capabilities. The present system can response to altered input keys, as well as authorize different password entries that are assembled from the the same set of keys.

Harmine (HM) in Fig. 1 was used in the present work.

It is a compound fabricated from a common tricyclic system, with dual hydrogen bonding sites at the adjacent positions. An indole and a pyridine ring act as proton donating and accepting sites respectively^25^. The existence of two aforesaid functional sites form the base for the dual sensing of anions and hence the dual channel keypad lock.

## Results

**Figure 2:**
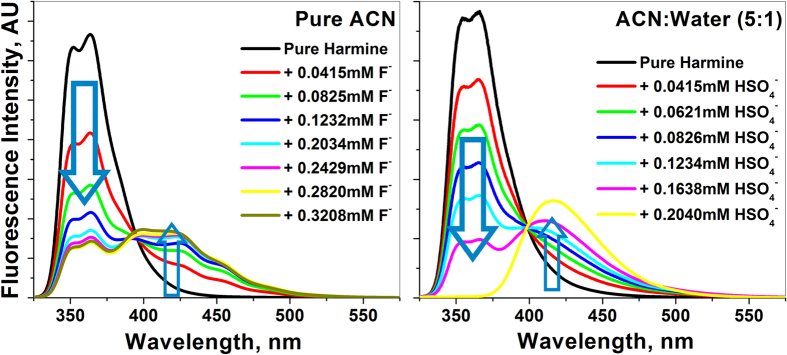
Changes in emission spectra of HM (**a**) in pure ACN with gradual addition of F^−^ ions (**b**) in 5:1 (v/v) ACN-water with gradual addition of HSO_4_^−^ ions; λ_exc_ = 300 nm; [HM] = 9.99 × 10^−4^ mM.

The optical spectra of HM in pure acetonitrile (ACN) medium shows two distinct bands at 300 nm and 365 nm for absorption and emission, respectively. Our host, HM displays an excellent chemosensory response both in absorption (Figures S1) and fluorescence upon addition of trace amount of fluoride ions (Fig. 2a). As we increase the fluoride ion concentration, unique new peaks around 330 nm in absorption and around 415 nm in fluorescence appear replacing existing bands showing distinct isobestic and isoemissive points at 288 nm and 397 nm, respectively (see SI). These absorption and fluorescence responses were successfully exploited, in the ratiometric way to detect and estimate the F^−^ anions up to 5 μM. To acquire the ratiometric response, we chose the fluorescence intensities at 365 nm and 415 nm. This ratiometric chemosensory response compares favorably to most of the known fluoride sensors^12^.

**Figure 3:**
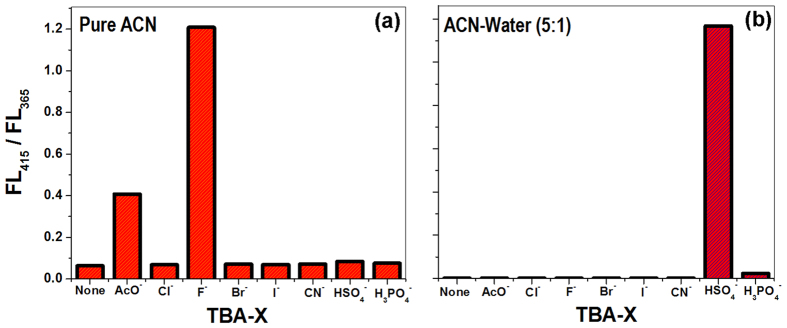
(**a**) Resultant ratio of fluorescence intensity measured at 415 and 365 nm for each anion (a) in ACN and (**b**) in 5:1 (v/v) ACN-water mixture.

The ratiometric sensing properties of HM towards HSO_4_^−^ anions were also tested. For this purpose we used the second channel i.e. the heterocyclic pyridine ring as binding site. To make it successful we used 5:1 (v:v) ACN-water mixture as solvent system to shut off the acidic pyrolic hydrogen channel. Presence of trace amount of HSO_4_^−^ modifies the absorption and emission spectra dramatically (Fig. 2b). To verify the anion specificity, similar experiments under identical conditions in both solvent systems were also carried out in the presence of other competitive anions (AcO^−^, Cl^−^, Br^−^, I^−^, CN^−^ & H_3_PO_4_^−^). The ratiometric optical response of HM was practically silent for these anions in spite of their presence in large excess as compared to F^−^ (Fig. 3a for pure ACN) and HSO_4_^−^ (Fig. 3b for ACN-water mixture).

Looking into the molecular mechanism behind sensing, we guess that titration of HM with F^−^ in pure ACN (Fig. 2a) resulted in quenching of fluorescence at 365 nm due to the formation of hydrogen bonded complex, thus acting as an “OFF” switch for 365 nm channel. However, the titration of the HM–F^−^ complex with HSO_4_^−^ resulted in the decomplexation with F^−^ ions and accompanied by a significant increase in fluorescence intensity at 365 nm, thus acting as an “ON” switch (Figure S3). On the contrary, in case of mixed solvents, upon titration with HSO_4_^−^ ions resulted in quenching of fluorescence at 365 nm, acting as an “OFF” switch (Fig. 2b). However, the titration of HM–HSO_4_^−^ system with F^−^ ion resulted in the restoration of the original fluorescence at 365 nm and acting as an “ON” switch (Figure S9). The reversibility of spectral response of HM was tested by carrying out several alternate cycles of titrations of HM with F^−^ followed by HSO_4_^−^ ions in pure ACN (Figure S4,5); and in reverse order (with HSO_4_^−^ followed by F^−^) in mixed solvents (Figure S10,11).

**Figure 4:**
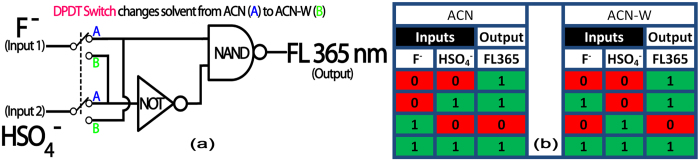
(**a**) Logic gate representation of the spectroscopic response of Harmine with sequential additions of F^−^/HSO_4_^−^ in ACN and (**b**) Corresponding truth table.

On the basis of reversible response of the sensor, a sequential IMPLY logic gate could be constructed using F^−^ and HSO_4_^−^ ions as the inputs and the fluorescence response at 365 nm as output. For pure ACN, one of the two inputs of a NAND gate is connected directly with the F^−^ input and the second one with the NOT gate output of the HSO_4_^−^, finally providing a perfect IMPLY gate output through fluorescence intensity changes at 365 nm. In case of ACN-Water mixture, the same IMPLY gate can be operated with the direct NAND and NOT input channels reversed between F^−^ and HSO_4_^−^. This is represented by the operation of a DPDT type switch in Fig. 4 that changes the solvent system from pure ACN to ACN-water mixture.

**Figure 5:**
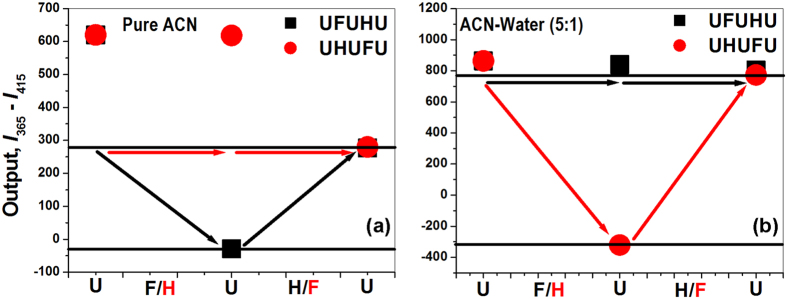
Password trajectories in pure ACN (**a**) and ACN-Water (5:1 (v/v)) (**b**) solvents. Variations of output “3” (*I*_365_ – *I*_415_) for each optical input, “U” throughout the full course of password entry.

The system was then further extended by including an additional fluorescence output channel at 415 nm (for both pure and mixed solvents) as the second output channel. Now, the sequential logic operations enable us to fabricate a binary logic gate which can work as a molecular keypad lock with three inputs (F^−^ ions, HSO_4_^−^ ions and 300 nm UV radiation, symbolized as “F”, “H” and “U”, respectively), and dual fluorescence outputs (at 365 nm & 415 nm). Finally, a third output has been considered as the difference between the fluorescence intensities at 365 nm (output 1) and 415 nm (output 2). The “OFF” state of the output “3” (=output “1” - output “2”) is the lowest value of the subtracted fluorescence intensity. The “ON” state is the highest (Fig. 5a,b). Depending upon the different combinations of three inputs, the output 3 then can switch between two emission states, “ON” and “OFF”. In pure acetonitrile, out of 27 possible input combinations (including any repeated entry), only the HFU and FHU combination turns “ON” the output 3 as shown in Fig. 5a. Similarly in case of mixed solvents only the above two combinations can turn “ON” output 3 (Fig. 5b), while the other combinations failed to do so. Hence, the system can be operated successfully with dual passwords but with similar output mode i.e. output “3” to authenticate the system in both pure and mixed solvents.

We intended to compose the system as a more complicated and intelligent device that can distinguish different users who are pre-registered with prior authority by harnessing the intrinsic merit of different passwords. So, we have planned to use the optical input (3^rd^ input) i.e. 300 nm light (U) thrice, making the password of five digit and much more complicated, thus, making it more secure. In this connection for the channel “1” (indole ring part, in pure ACN solvents), first we used U, F, followed by U, then H and finally U again, creating the password UFUHU and for the other possibility first we used U, H, followed by U, then F and finally U again, as a result password generated was UHUFU. Unlike simple chemical password entry which may be optically monitored only once at the end, here the whole course i.e. each and every step of the password entry will be under complete optical monitoring. This is similar to the measurement for the change of a thermodynamic path function that is not only dependent on the initial and final states, but, the adapted path needs to be considered. In case of UFUHU password entry in pure ACN when the first three inputs (U, F & U; UFU) are inserted sequentially then instantaneously one would observe the fluorescence intensity diminished at 365 nm and augmented at 415 nm (Figs 3a and 5a). Finally, as the last two inputs (H & U; HU) are introduced, the fluorescence intensity of output 3 changes significantly and ultimately brings it at “ON” state. While dealing with UHUFU password in pure ACN, there is no change of spectral pattern (Fig. 5a) when first three inputs (U, H & U; UHU) are provided. However, when next two inputs (F & U; FU) were inserted, it shows the fluorescence at output “3” (Fig. 5a). For the sake of illustration, we have presented a conceptual password trajectory monitoring system that correlates the fluorescence intensities at 365 & 415 nm for each input case and connects the resultant subtracted intensities with each “U” input, presenting a trajectory (Fig. 5a,b) for each full password entry in each solvent.

The most interesting feature of the present concept is that two passwords (UFUHU & UHUFU) generate same final optical state, but, still they are distinguishable since they follow totally different optical trajectories. These exclusive features of tracking the optical trajectory enable our developed device to distinguish between the authorized and unauthorized password providers. This feature is also equally available when the second channel (i.e. for heterocyclic pyridine part) works as hydrogen bonding sites in the mixed solvent system. Interestingly, as in the case of pure solvent, here too the above two passwords (UHUFU and UFUHU) will work based on completely different optical output trajectory recognition and follow a fully different (reverse) optical output trajectories (Fig. 5b).

**Figure 6:**
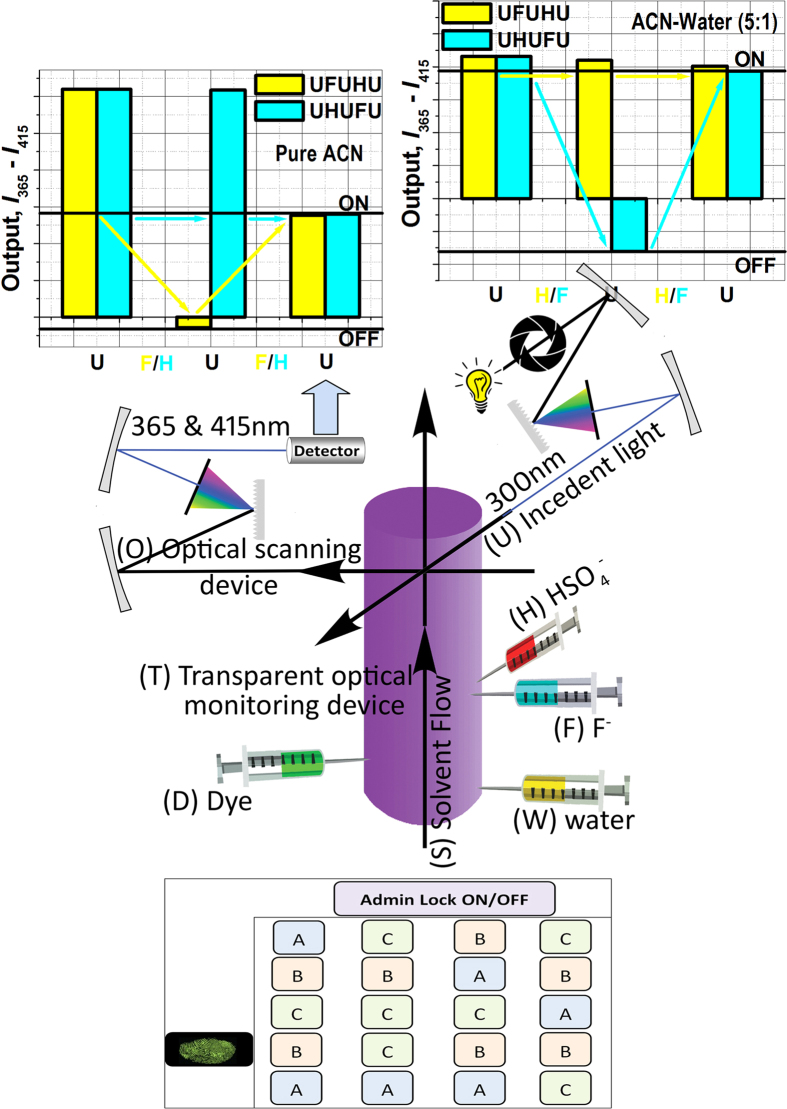
Operational mechanism of the opto-chemical lock.

It is important to note that there can be 100 different possible combinations with a simple two-figure fundamental password, with the numerical digits from 0 to 9. Further, 676 different probable combination are possible using individual letters from A to Z. This can be made possible by some easy and suitable computer programming which could relate each input corresponding to one alphabet or one numerical digit. Furthermore, 1296 different possible combinations are feasible while picking up a two-figure password composed with both the numerical digits (from 0 to 9) and the alphabets (from A to Z). Our design supports two five-figure fundamental passwords (UFUHU and UHUFU) for each solvent and each pattern recognition. So enhancement of security level in this type of device is easily understandable through picking up of a working password from such a huge number of alphanumeric combinations. Finally, we have designed a device to execute the idea of an programmable opto-chemical protection system following the password trajectories (Fig. 6). The proposed operational mechanism of the pure opto-chemical lock is as follows:

The main structure of the opto-chemical lock is based on a transparent quartz cylindrical flow system (T) through which the acetonitrile solvent is flowing at a constant flow rate (S). Four automated piston driven injectors filled with water (W) and solutions of HM (D), F^−^ (F), HSO_4_^−^ (H) are attached sequentially at different positions from bottom to top with the cylinder along the direction of the flow of solvent. After a suitable hydrodynamic distance for mixing above the injectors, the optical devices for 300 nm source with a shutter arrangement (U) and a simultaneous dual channel (365 nm & 415 nm) fluorescence detectors (O) are placed at right angles with source for monitoring fluorescence intensities of HM at 365 nm & 415 nm. The injectors are activated by corresponding input devices (buttons on the input panel). The “Admin Lock ON/OFF” button activates the injector ‘D’ that injects the DMF solution of HM at a constant rate continuously for a preset time (displayed on a admin programmable countdown timer) during which the lock remains activated and an authorized user can access the lock.

After the preset time is over (the countdown timer stops), the injection of the dye stops and the lock becomes unresponsive. Now an authorized user is authenticated by a biometric verification (like fingerprint or retina scanning) which is controlled by a biometric databank pre-configured for a group of authorized users. This biometric scanning of an authorized user activates all optical mechanism including the 300 nm light source. Finally, a recorder records the intensity differences between these two channels (Output 3) with respect to time or the entries on the user keypad. After user authentication, an authorized user can use the user keypad for a limited time (limited by the countdown timer). There are buttons of A, B and C type arbitrarily arranged on the keypad. It is not disclosed that which buttons are assigned to injectors (F^−^/HSO_4_^−^) and to the shutter opener for 300 nm incident light. It is pre-programmed that the injectors may be activated only once and during the countdown timer runs, whereas, the 300 nm shutter may be opened several times. Now, one can sequentially understand the operational mechanism of the lock as follows:

i) “Admin Lock ON/OFF” button for Administrator: Administrator activates the lock by pressing this switch that injects HM (D) to the transparent flow system (T) where incident monochromatic light of 300 nm (U) can interact with HM.

ii) Biometric “Track” button for user: Through this input device, an authorized user biometrically activates or initiates the optical scanning device (O) that records the optical output trajectory (output “3”). The user identification is authenticated at this stage, since only registered users are able to access the lock.

iii) Main “Keypad” for user: Within a limited time scale (shown on the countdown display) the user can press some/any button(s) on the user keypad. A proper password entry with a proper sequence (say, CACBC meaning UFUHU) by the user respectively and sequentially opens the 300 nm shutter, then activates the F^−^ injector, followed by the opening of the 300 nm shutter, activating the HSO_4_^−^ injector and again opening of the 300 nm shutter. This sequential operation of the injectors and the shutter generates a trajectory graph on the recorder. Matching of this trajectory recording finally opens the lock.

iv). Lock “Reconfiguration”: Interestingly, the administrator can reconfigure the lock by pressing a secret button that activates the injector ‘W’ (not displayed on keypad) which continuously injects water with a preset speed so that the solvent flowing through the channel becomes 5:1 (v/v) mixture of acetonitrile-water. Now the password to open the lock fully changes and becomes UHUFU i.e. CBCAC on the user keypad.

## Methods

Harmine procured from Aldrich Chemicals (USA) was purified by recrystallization from ethanol. All anions were purchased from Sigma-Aldrich in the form of tetrabutyl ammonim salts and used without any further purification. All solvents used in the present experiments were spectroscopic grade from Spectrochem, India. Shimadzu 1600 spectrophotometer and Hitachi F4600 spectrofluorometer were used for the absorption and emission spectral studies, respectively.

Stock solutions of 1.2 mM HM in DMF, 10 mM TBAB in acetonitrile and 10 mM TBAF in acetonitrile were prepared. Other stock solutions of similar anions (tetrabutylammonim salts) were also prepared accordingly in acetonitrile. About 2400 μL of solvent (pure acetonitrile or 5:1 ACN-water mixture) was taken first in a teflon stoppered quartz cuvette, then 2 μL HM was added, stirred on a magnetic stirrer, thermally equilibrated to 300 K and exposed to fluorescence measurements. 300 nm light was used for excitation. Then appropriate volume fractions of the respective tetrabutylammonim salt stock solutions of bisulfate or/and fluoride or/and others were added as required to the final mixture, stirred well and the fluorescence spectra were recorded. These steps were repeated until spectral saturation was observed.

## Conclusions

In conclusion, HM, a commercial fluorescent dye provides a simple hitherto unexplored class of highly selective and efficient fluorescent sensor molecule for fluoride and hydrogen sulphate ions, enabling us to fabricate an opto-chemical molecular lock with resettable and reconfigurable password. Conventional chemical password entry implies optical monitoring after full password entry. The novelty of the present idea lies in the simultaneous dual channel monitoring over the whole course of multiple entries of password elements. The system may get blocked or stopped after accepting wrong entries at any step. The whole wall of the lock concept is made of combinations of optical and chemical building blocks, i.e., opto-chemical in true sense. Still there are considerable engineering and technical requirements to put life to the proposed idea and finally fabricate a device, ready to use.

## Additional Information

**How to cite this article:** Majumdar, T. *et al*. A Strategic Design of an Opto-Chemical Security Device with Resettable and Reconfigurable Password Based Upon Dual Channel Two-in-One Chemosensor Molecule. *Sci. Rep.* **7**, 42811; doi: 10.1038/srep42811 (2017).

**Publisher's note:** Springer Nature remains neutral with regard to jurisdictional claims in published maps and institutional affiliations.

## Supplementary Material

Supplementary Dataset 1

## Figures and Tables

**Figure 1:**
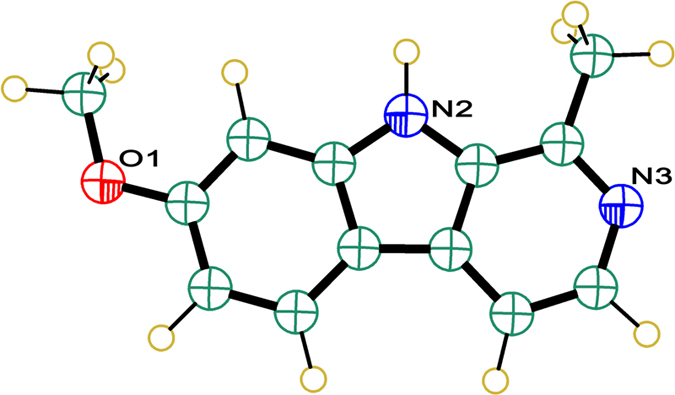
Structure of HM.
