# Supplementary material for: A Strategic Design of an Opto-Chemical Security Device with Resettable and Reconfigurable Password Based Upon Dual Channel Two-in-One Chemosensor Molecule
Source: Sci Rep. 2017 Feb 20;7:42811. doi: 10.1038/srep42811 (PMC5316941; doi:10.1038/srep42811)
Supplement: Supplementary Dataset 1 [file srep42811-s1.doc]

**Title: A Strategic Design of an Opto-Chemical Security Device with Resettable and Reconfigurable Password Based Upon Dual Channel Two-in-One Chemosensor Molecule**

**Authors and affiliations:**

Tapas Majumdar^a^ , Basudeb Haldar^b^ and Arabinda Mallick*^c^

^a^Department of Chemistry, University of Kalyani, Nadia, West Bengal-741222, India

^b^Department of Chemistry, Vivekananda Mahavidyalaya, Burdwan, Westbengal-713103, India

^c^Department of Chemistry, Kashipur Michael Madhusudan Mahavidyalaya, Purulia, Westbengal-723132, India

*Corresponding author, E-mail: AM: [ampcju@yahoo.co.in](mailto:ampcju@yahoo.co.in)

**Supplementary Information:**

**A. Pure ACN**

**1. Effects of F^-^ and HSO_4_^-^ on the UV-Vis spectra of HM in pure ACN**


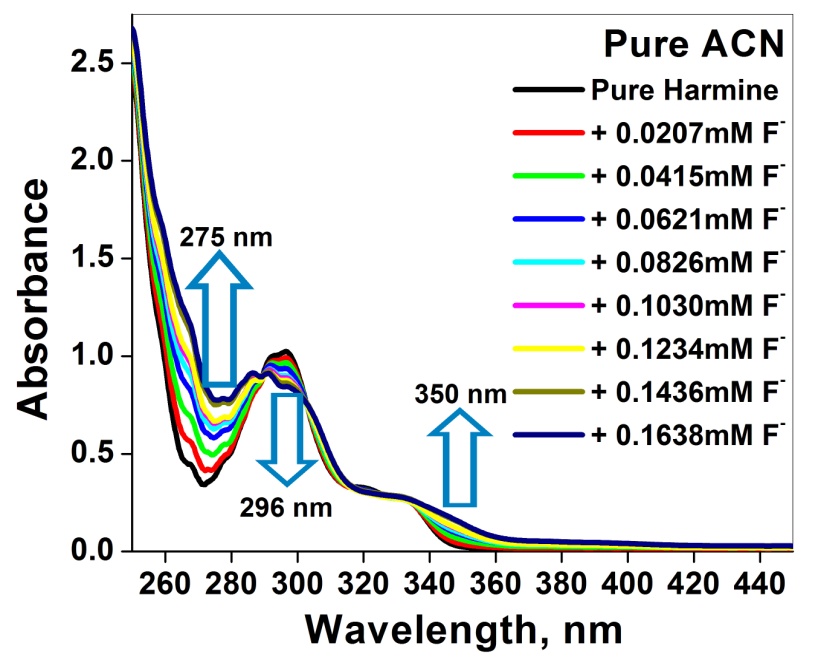
(a). Effect of F^-^ on absorption spectra of HM.

Figure S1: Absorption spectra of HM (1×10^-3^ mM) in pure ACN in the presence of various amounts of F^-^ anions.

(b). Effect of HSO_4_^-^ on absorption spectra of HM-F^-^ complex.


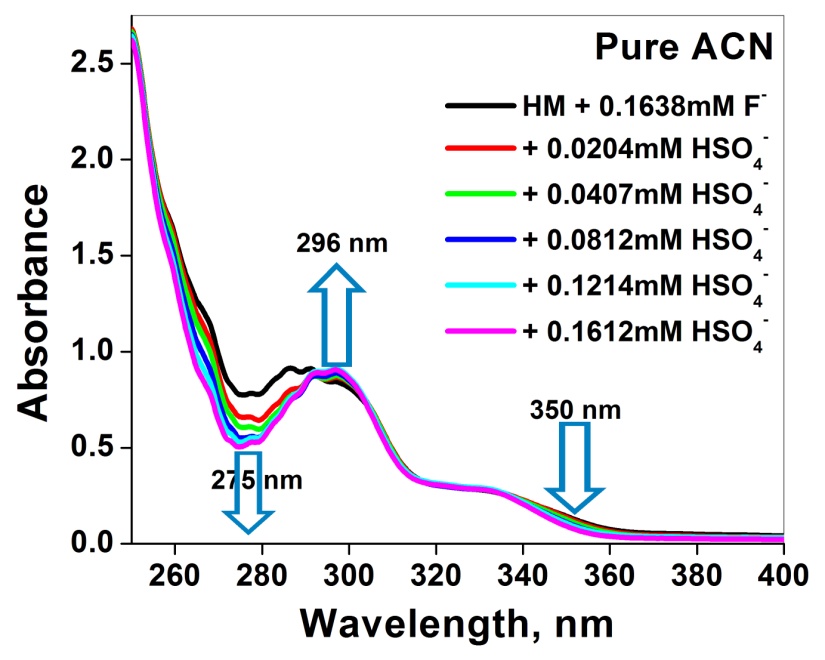


Figure S2: Absorption spectra of HM-F^-^ complex in pure ACN with gradual addition of HSO_4_^-^ anions.

**2. Emission spectra of HM in pure ACN solvent:** Effect of HSO_4_^-^ on emission spectra of Harmine-F^-^ complex


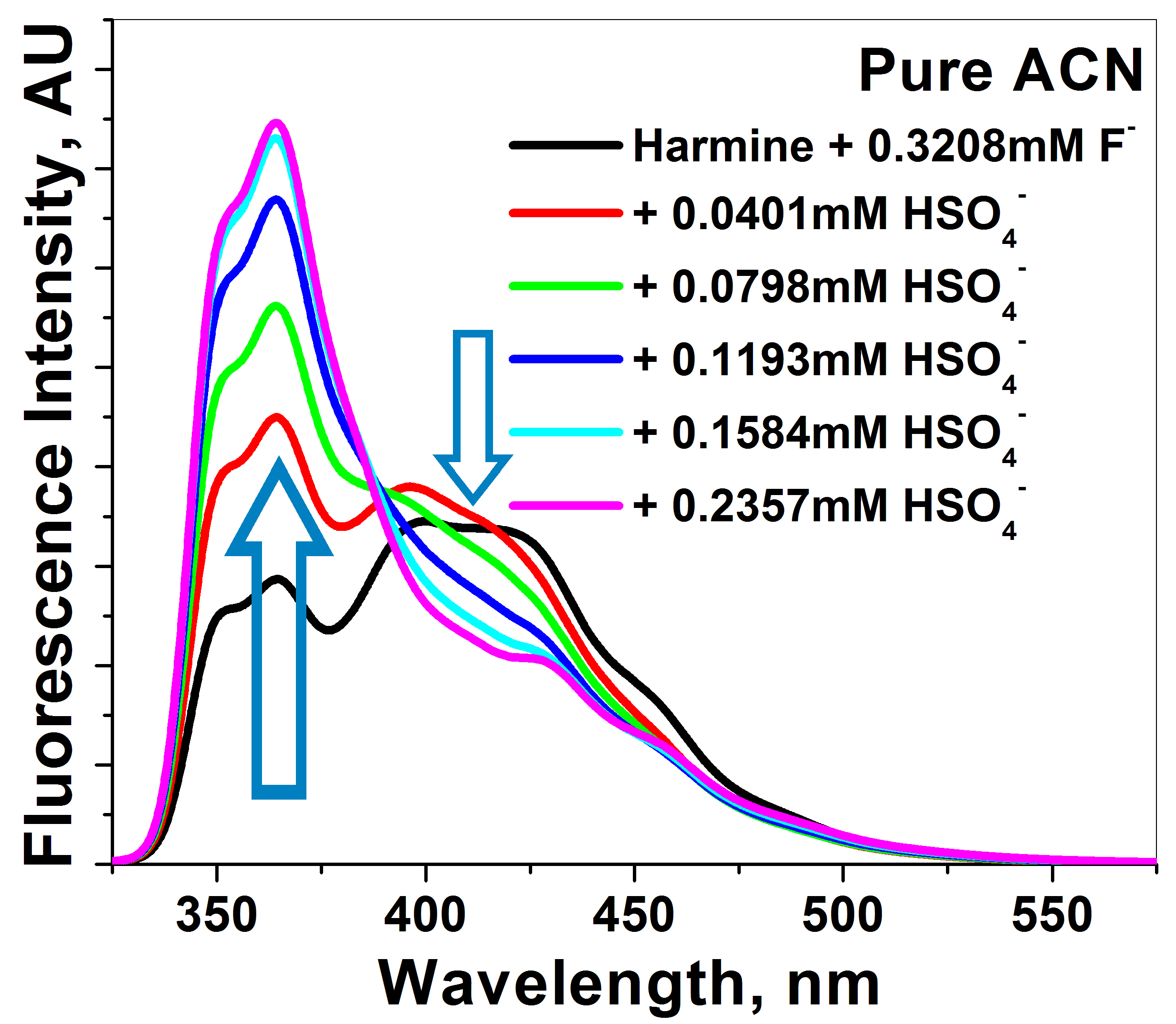


Figure S3: Emission spectra of HM-F^-^ complex in pure ACN with gradual addition of HSO_4_^-^ anions.

**3. Reversibility of the ratiometric response:**

(a). Monitoring at 365 nm


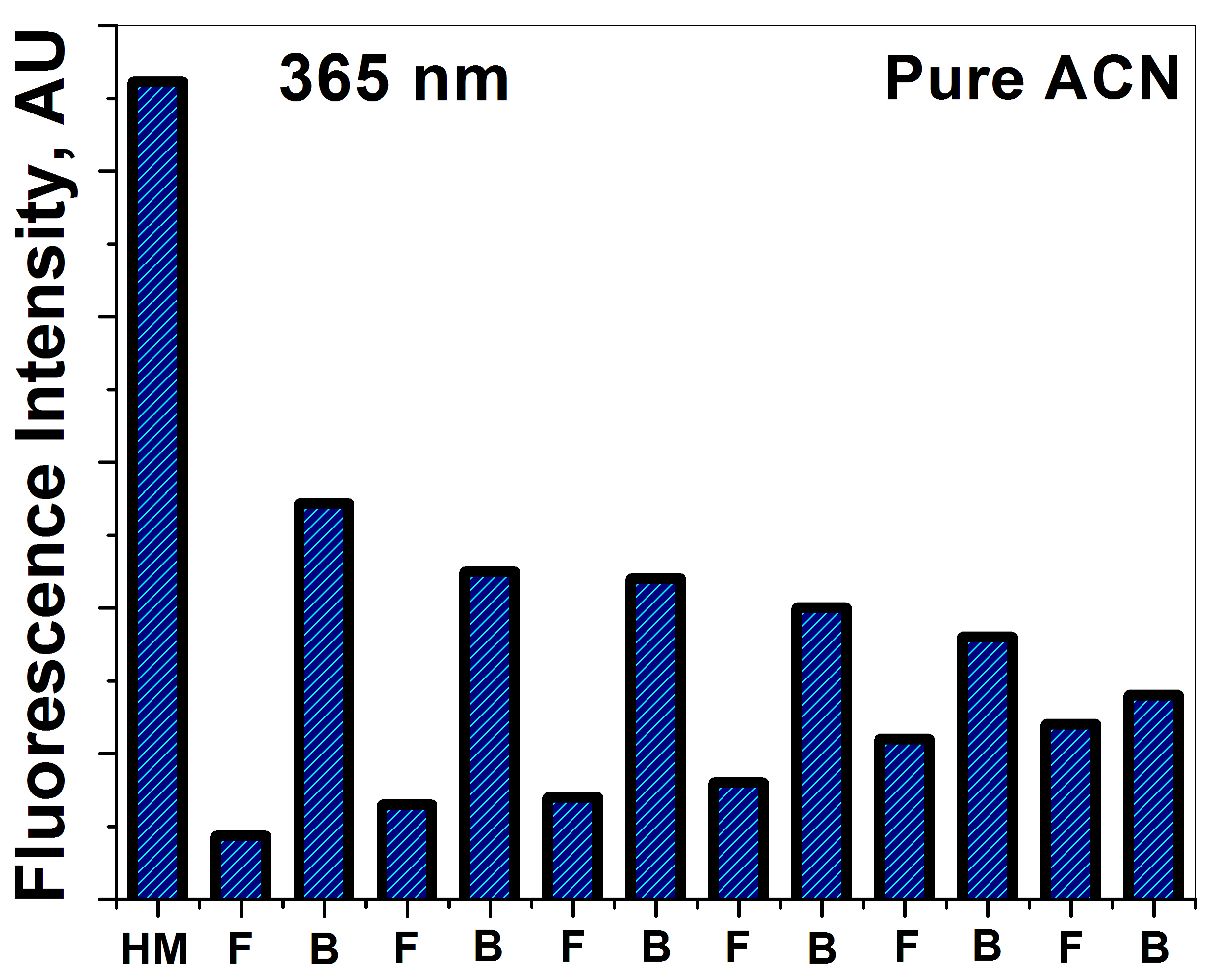


Figure S4: The bar graph of the fluorescence intensity of HM at 365 nm showing how reversibly HM responses to the alternate addition of F^‑^ and HSO_4_^-^ ions.

(b). Monitoring at 415 nm

**
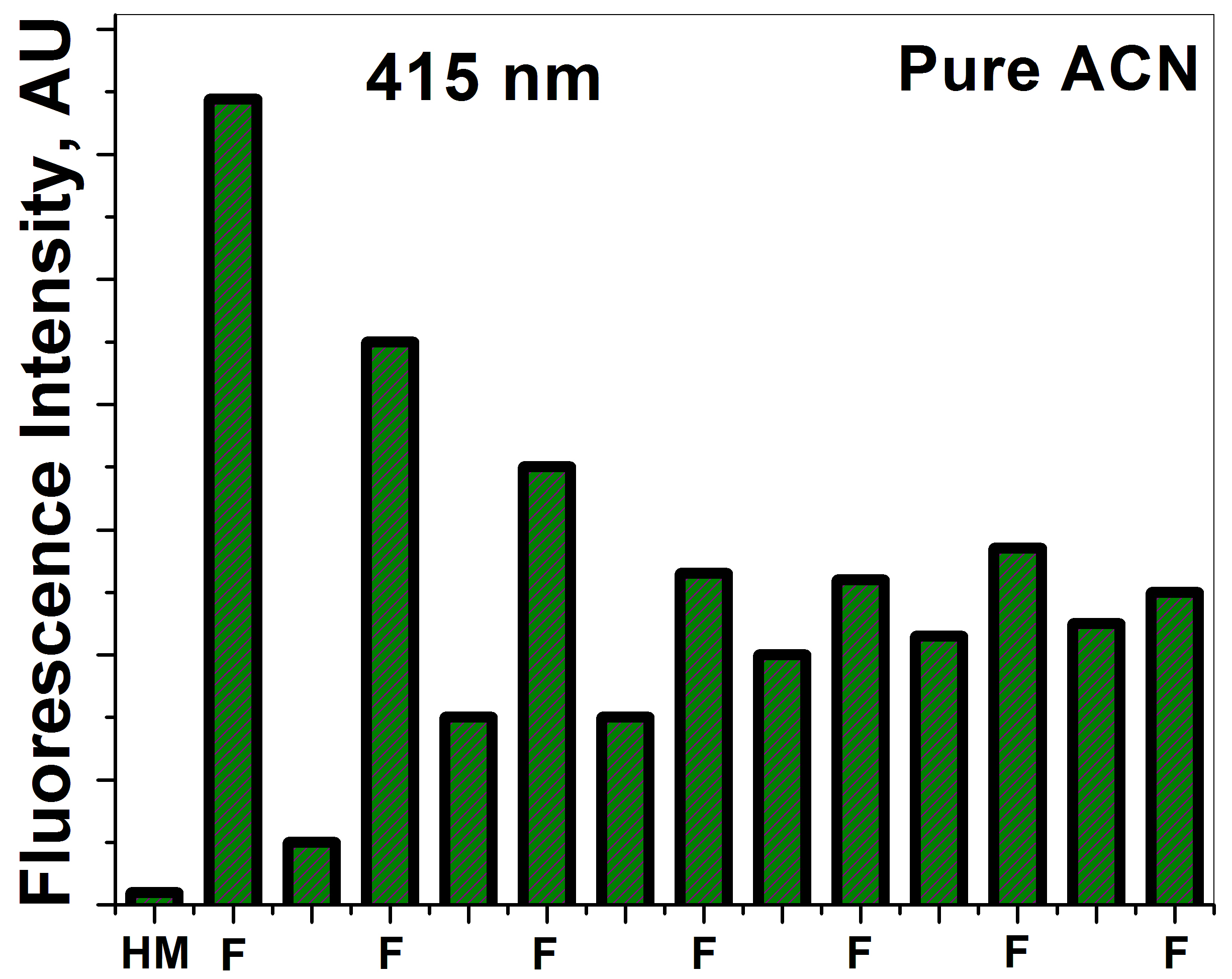
**

Figure S5: The bar graph of the fluorescence intensity of HM at 415 nm showing how reversibly HM responses to the alternate addition of F^‑^ and HSO_4_^-^ ions.

**B. 5:1 (v/v) ACN-Water Mixture:**

**1. UV-Vis spectra of HM in 5:1 (v/v) ACN- Water mixed solvent**


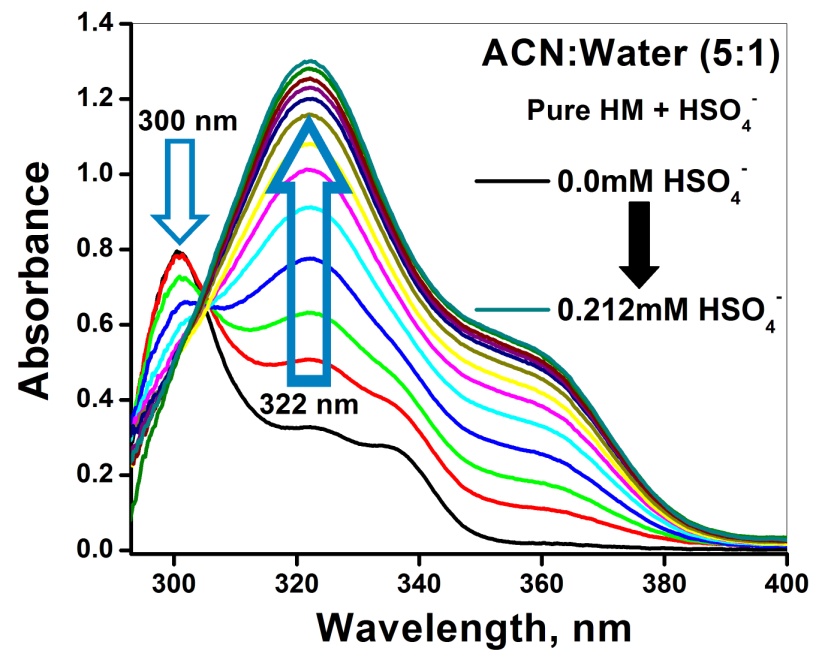
(a). Effect of HSO_4_^-^ on absorption spectra of HM.

Figure S6: Absorption spectra of HM in 5:1 (v/v) ACN-Water mixture in the presence of various amounts of HSO_4_^-^ anions.


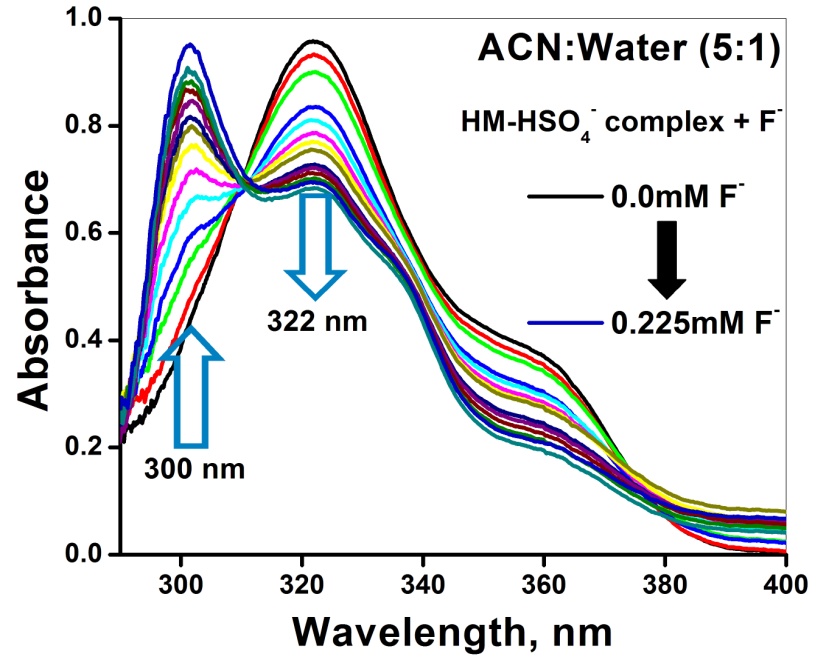
(b). Effect of F^-^ on absorption spectra of HM-HSO_4_^-^ complex.

Figure S7: Absorption spectra of HM-F^‑^ complex in 5:1 (v/v) ACN-Water mixture in the presence of various amounts of F^-^ anions..

**2. Solvatochromic Effect: Effect of water on the fluorescence spectra of HM in ACN**


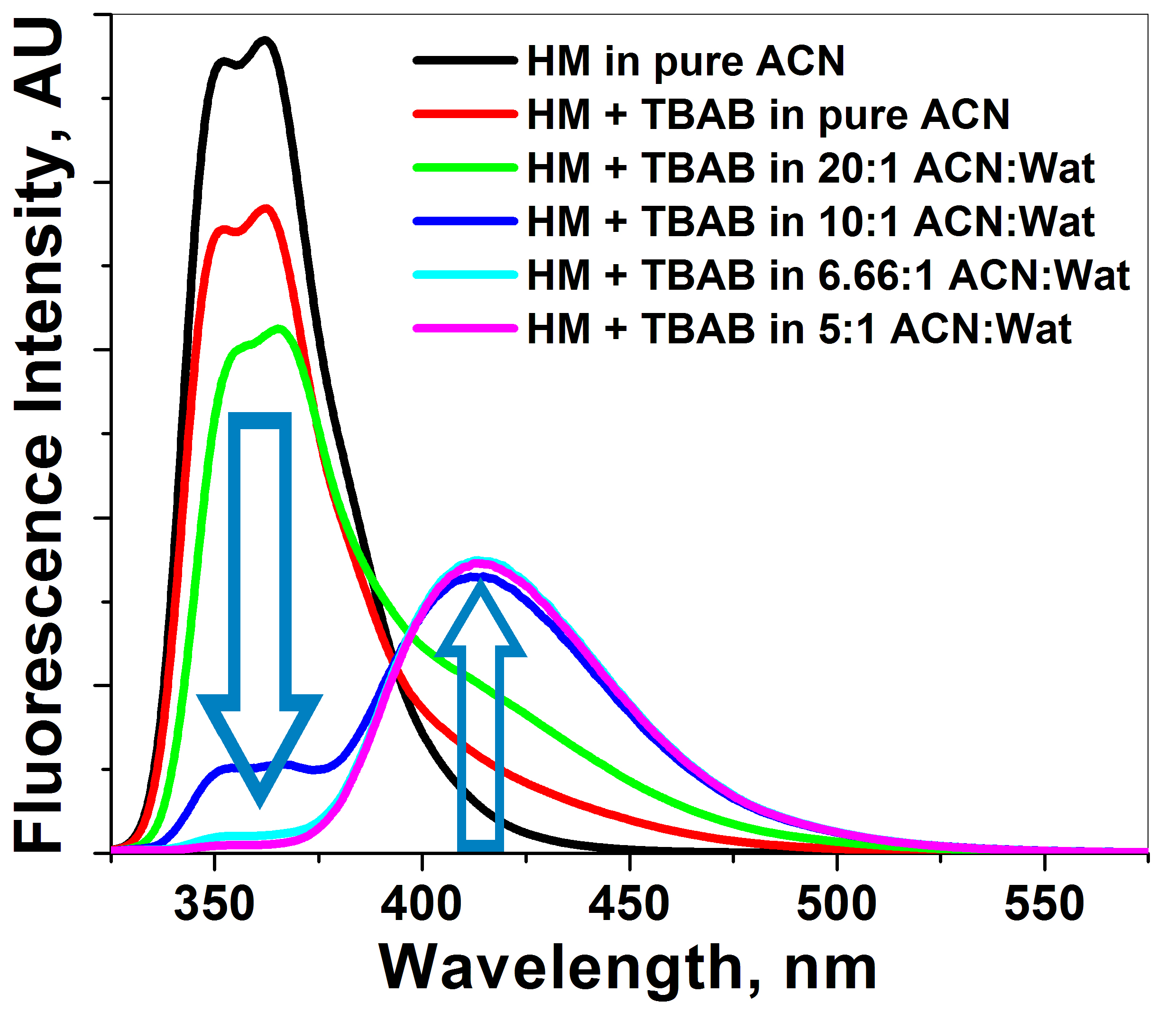


Figure S8: Fluorescence spectra of HM in pure ACN in presence of trace amount of HSO_4_^-^ anions and how it changes in presence of water fractions.

**3. Emission spectra of HM in 5:1 (v/v) ACN- Water mixed solvent:** Effect of F^-^ on emission spectra of HM-HSO_4_^-^ complex

**
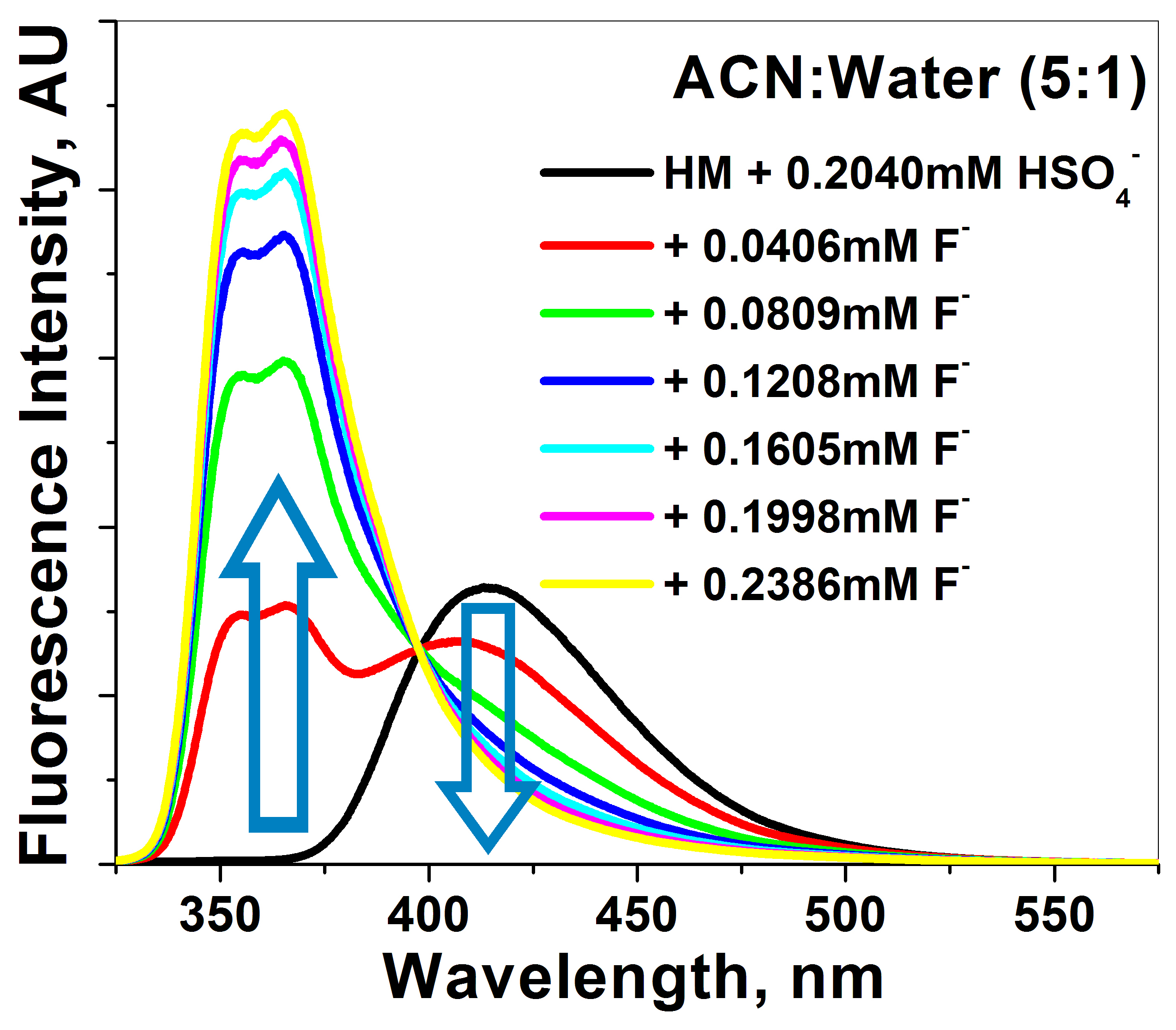
**

Figure S9: Fluorescence spectra of HM-HSO_4_^-^ complex in 5:1 (v/v) ACN-Water mixture in presence of increasing amounts of F^-^ anions.

**4. Reversibility of the ratiometric response:**

(a). Monitoring at 365 nm


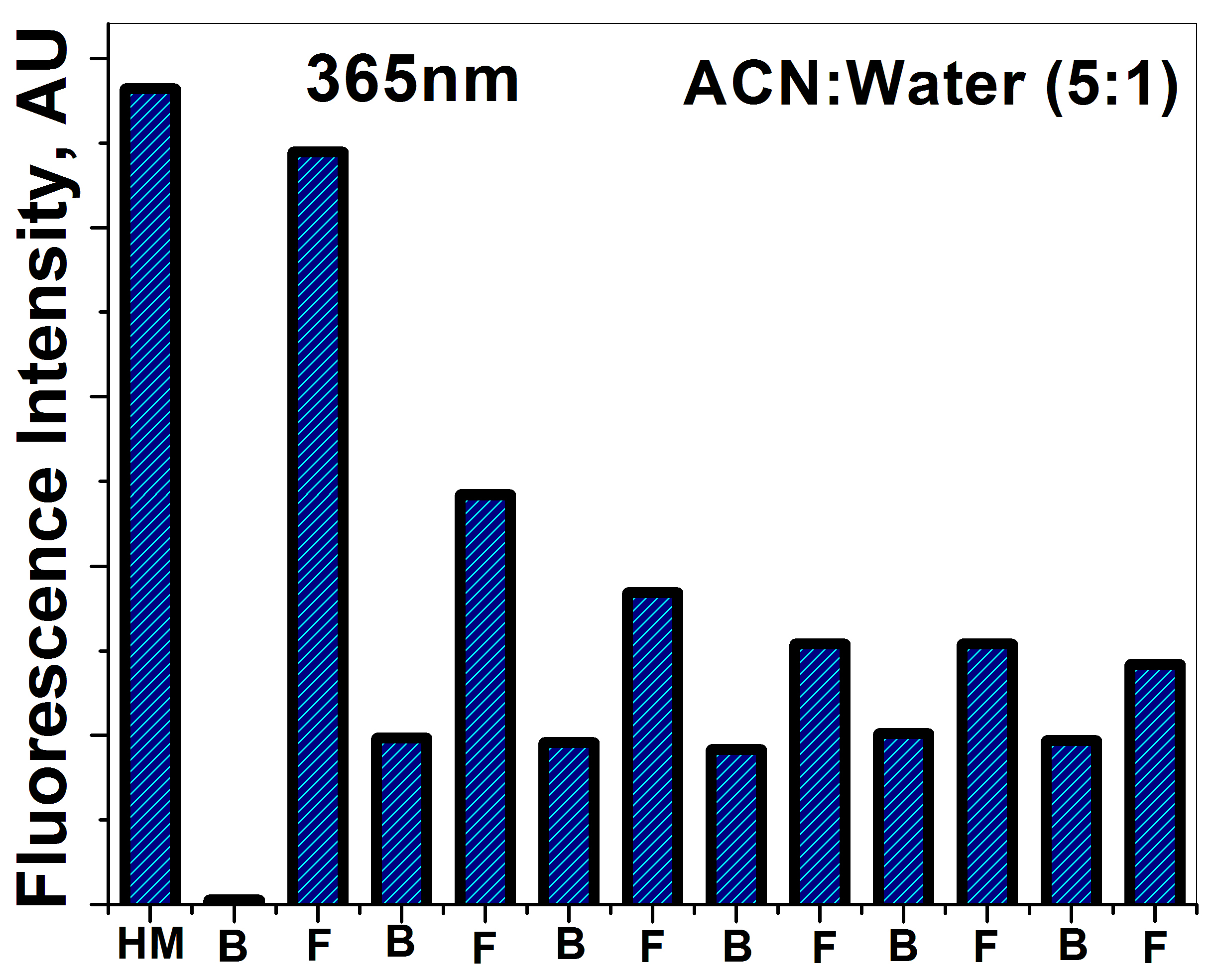


Figure S10: Fluorescence intensity bars at 365 nm of HM showing how reversibly HM responses to the alternate addition of HSO_4_^-^ and F^‑^ ions.


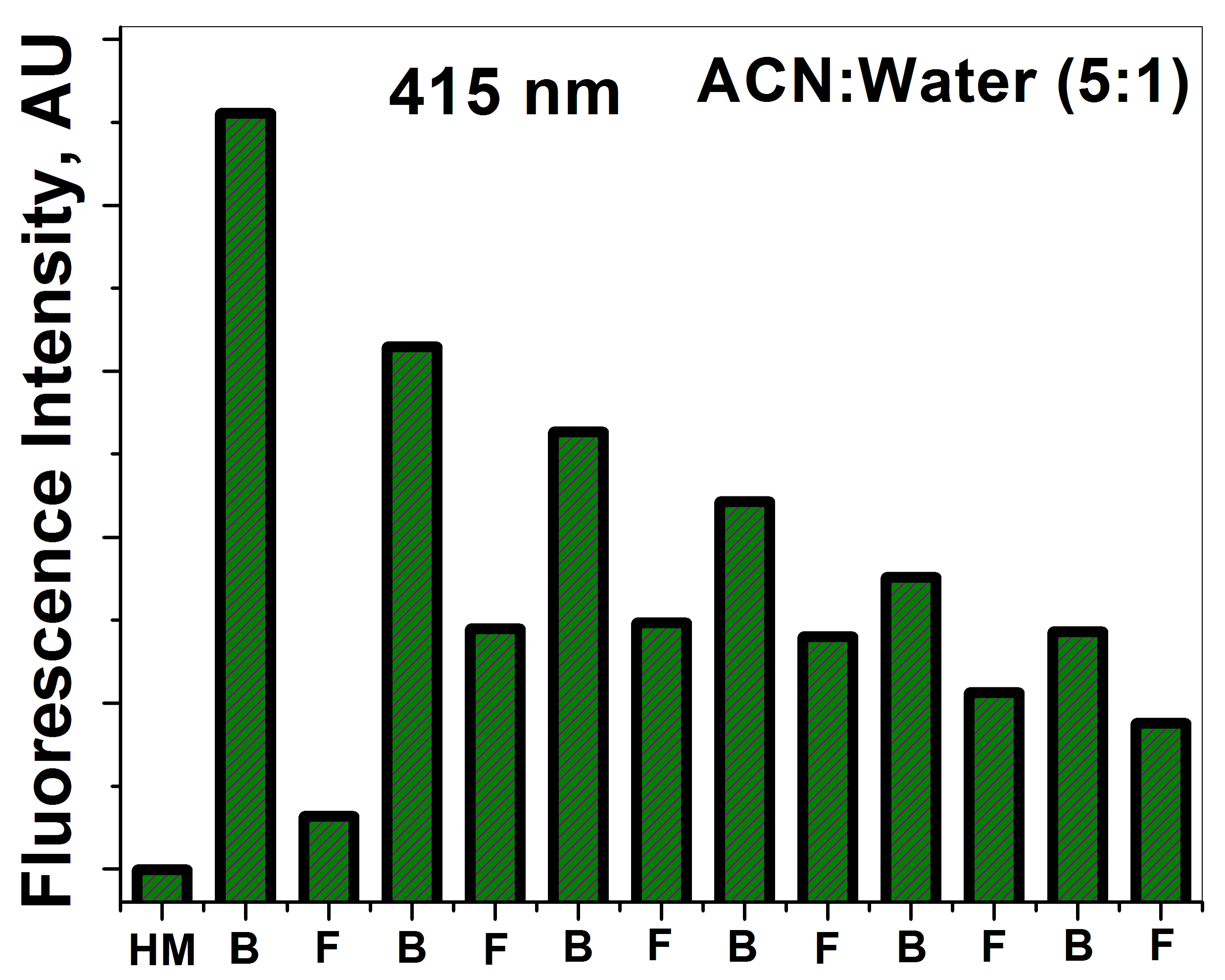
(b). Monitoring at 415 nm

Figure S11: Fluorescence intensity bars at 415 nm of HM showing how reversibly HM responses to the alternate addition of HSO_4_^-^ and F^‑^ ions.

**Experimental procedure for reversibility, reusability, reset-ability, reconfigure-ability of the lock, in addition to demonstration of the blocking and stopping upon wrong entry.**

We have carried out total experiments in the static system of quartz cuvettes during steady state absorption and emission measurements. We performed experiments in the pure acetonitrile solvent and 5:1 (v/v) ACN-Water mixed solvents. Moreover, in the static system itself we have found the 3-4 cycle reversibility in two solvent systems. On the basis of the sequential reversible ratiometric response of HM over interaction with F^-^ and HSO_4_^-^ respectively in the pure ACN and in the reverse order in 5:1 (v/v) ACN-Water mixed solvents, we designed a conceptual opto-chemical device. This conceptual device fully based on a flow system rather on a static cuvette system.

**Reversibility & reusability:**

The opto-chemical device is designed on a flow system where the pure acetonitrile is continuously and constantly flowing simply like the HPLC system. Now the "Reversibility" arises out of the flow system alone. The optical instrumentation (the 300 nm source and the 365 nm & 415 nm detectors) monitors the fluorescence outputs. Until the ACN is flowing alone, the detectors show continuous zero fluorescence intensity. As long as the injectors injects the HM, F^-^, HSO_4_^-^ and the water, the detectors show the fluorescence intensity variations. As all the injectors stop injecting the aforementioned chemicals, there is only the pure ACN solvent keeps flowing and the fluorescence outputs show zero intensity as before. Again if HM, anions and water are injected, the recording device detects signals. Again as the injectors start injecting, there are optical signals detected. During this switching between the working and not-working state of the device, the ACN solvent keeps flowing constantly. So no one needs to attend the system. Hence the system is fully reversible and reusable.

**Reset-ability & reconfigure-ability:**

The electronic keypad works as the interface device between the humans (administrator and users) and the opto-chemical device. A programmable computer software or an electronic device links the electronic keypad and the electro-mechanical parts (injectors and source shutter) of the device and also the signal detectors. Also there must be a database recording, storage and matching program in the computer that finally links the signal detectors and finally the lock that is intended to be opened and closed. The keys on the keypad must link the shutter and the injectors to activate them. Now which key activates which components, is controlled by a computer program just like public and privet encryption keys in information technology. These digital keys may be easily managed by, for example, a enterprise server system through networking.

Like every digital number-pad locks and pattern locks, the working keys of this opto-chemical lock are easily customizable and also reset-able to factory settings, but strictly through administrator authentication. The term "reconfigurable" here represents the feature "customizable keys" through administrator authentication on the working software platform.

**Blocking and stopping upon wrong entry:**

The opto-chemical lock runs on a software platform. During operation i.e. entry of keys, the output signal at every entry is simultaneously recorded and matched with the stored database. During a full password entry, if at any sequential step (3rd or 4th) does not match with the trajectory database; the software may immediately deactivate the keypad until further reactivation by the administrator. This feature blocks an user to enter wrong password and stops the device accepting next wrong keys making the device more secure. Because of this unique feature, an user who does not know the right password, cannot try multiple passwords randomly to find a valid password. This is very similar to a common feature of digital devices that permanently locks or resets or bricks the device over a preset number of wrong password entries. Our design will prevent users to enter even a single wrong key and accept only the fully correct password. Upon a wrong key entry, the current user could not complete the full password entry. Also the user could not try a second time without administrator's concern.

**The discussions involving solvatochromic effect**

"In the pure acetonitrile medium, i.e. in a pure polar but non-protic solvent environment, there no chance of protonation or deprotonation for HM. So in the emission spectrum of HM in pure ACN, we see only the neutral band at 365 nm. But in presence of trace amount of highly basic F^-^, the HM gets deprotonated through the pyrolic proton in the ground state as well as in the excited state. This deprotonation of HM by F^-^ is well reflected in the raising of the cationic bands and simultaneous diminished neutral bands in the steady state absorption and emission spectra recorded with gradual addition of F^-^. Hence the pyrrolic -N-H is the only active hydrogen bonding site as acceptor.

But the scenario changes completely in the 5:1 (v/v) ACN-Water mixed solvent. We performed fluorescence measurements of pure HM in pure ACN and we saw only the pure neutral band. Little HSO_4_^-^ addition caused a little significant change in the emission spectra. But when we added volume fraction of water in the mixture, we noticed significant shift of band from neutral to cationic (Figure S8). At a 5:1 (v/v) ACN-Water mixture we observed the total disappearing of the neutral band and appearance of a full new cationic band (Figure S8). These observations makes us conclude that in the 5:1 (v/v) ACN-Water mixed solvent, the HSO_4_^-^ ions best interact with HM through strong hydrogen bonding. And this is only possible at the pyridinic-N center. So we assume that in presence of trace amount of water, the pyrrolic protons in HM are fully engaged with the water molecules in hydrogen bonding and they are no longer available for interacting with the F^-^ (Figure 3b). Hence in the mixed solvent, the active hydrogen bonding site is the pyridine N as a proton acceptor and not the pyrrolic -N-H.
